# Best-Laid Plans: Can a “Life-Plan” Improve the Concordance of Kidney Disease Care with Patient Preferences?

**DOI:** 10.34067/KID.0000000000000139

**Published:** 2023-06-29

**Authors:** Megan Urbanski, Laura C. Plantinga

**Affiliations:** 1 Department of Surgery, Emory University, Atlanta, Georgia; 2 Department of Medicine, Emory University, Atlanta, Georgia

**Keywords:** CKD, clinical nephrology, ESKD, geriatric nephrology, patient satisfaction, patient-centered care

The implementation of individualized approaches, incorporating information about patients' preferences for care as well as prognostic and other factors, has been a significant challenge in US dialysis care.^1^ “One size fits all” approaches have dominated dialysis care in the pay-for-performance era^2^: For example, the Fistula First initiative^3^ was predicated on the assumption that an arteriovenous fistula was best for all in-center hemodialysis patients. To address this issue, the National Kidney Foundation recently expanded their Kidney Disease Outcomes Quality Initiative vascular access clinical practice guidelines to include a universal recommendation for an ESKD Life-Plan. The ESKD Life-Plan promotes shared decision-making (SDM) among patients and their dialysis care team as they strategize treatment plans for a lifetime with kidney disease and “specifically considers the patients' current medical situation, current and future life goals, preferences, social support, functional status, and logistics and other practical feasibilities.”^4^ The recommendation states that the ESKD Life-Plan should be established for those with progressive CKD, estimated glomerular filtration rate <20 ml/min per 1.73 m^2^, or ESKD and that this Life-Plan should be reviewed, updated, and documented in the patient's medical record at least annually and more frequently as clinically indicated.^4^ The ESKD Life-Plan has obvious relevance beyond vascular access management, yet the extent of its uptake—and incorporation of patient preferences generally—into the spectrum of care for patients with ESKD and pre-ESKD CKD dialysis remains underexplored.

**Figure 1:**
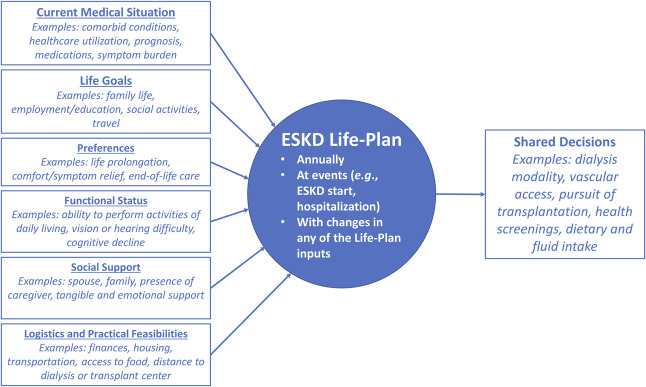
Generalized application of the ESKD Life-Plan in CKD and ESKD care.

In this issue of *Kidney360*, Kazi *et al.*^5^ address this gap by exploring treatment plan preference (dichotomized as extending life versus relieving pain and discomfort), agreement that the current treatment plan meets these preferences, and SDM among 213 hospitalized patients on maintenance hemodialysis, using a cross-sectional survey. Overall, they found that 58% preferred comfort care over life-extending care, while 42% disagreed that they were receiving care in concordance with their preference. Those preferring comfort versus life-extending care were 85% less likely to report preference-concordant care; those with higher SDM scores were more likely to report preference-concordant care (2% higher likelihood with each point on the 0–100 SDM scale). These findings, while disappointing, are unsurprising, given pervasive upstream barriers to preference-concordant CKD care. Patients along the CKD continuum often lack the requisite information to have clear preferences and make informed decisions about their care and treatment. The authors also assessed participants' perceptions of SDM regarding the decision to start dialysis. However, existing evidence has largely suggested that patients rarely feel they have a choice when it comes to initiating dialysis.^6^ Although SDM is often regarded as a best practice for complex medical decision-making, it may not be enough to help patients make treatment decisions that align with their personal values and wishes, as well as their cultural and religious beliefs. The ESKD Life-Plan, generalized to CKD and ESKD care (Figure 1), could provide a roadmap for patient-driven care team discussions of goals of care, which could ultimately improve the preference concordance of care delivered in this population.

In their study, Kazi *et al.*^5^ surveyed patients during a hospital stay, but the cause of hospitalization, length of hospital stay at the time of survey administration, and existing co-morbidities were unknown. Furthermore, the measure of preference-concordant care did not distinguish between preferences for dialysis care and the care being received at the time of hospitalization, which limits the interpretation of the study's findings, as the authors acknowledge. These contextual factors (*e.g.*, care-related decisions to be made, increasing age, changes in social or socioeconomic status, new-onset comorbidity or disability, disease progression, and acuity of illness) likely influence patient preferences and highlight the need for continuous discussions regarding goals of care that are initiated by providers. Thus, a generalized ESKD Life-Plan would not be static, but rather responsive to these contextual changes (Figure 1).

To engage in these conversations meaningfully and at every care juncture, providers likely need additional support and resources such as communication skills training and improved availability of interdisciplinary team members to offset time constraints. Better means of coordinating care across settings is also needed. Even if the dialysis care team providers work with the patient to optimize the elicitation and documentation of patient preferences, including but not limited to advance care planning, these preferences may not translate to other settings in which patients receiving dialysis are treated. To illustrate, Kazi *et al.*^5^ examined patients in the hospital setting. In the fragmented US health care system, dialysis clinics and hospitals do not frequently share health records or include information in health information exchanges (which may not be used by the dialysis clinic or hospital). Thus, it is unlikely that dialysis clinic providers frequently share documentation of preferences with hospital providers; conversely, it is equally unlikely that hospital providers elicit changes in preferences related to hospitalization events or communicate these changes to the dialysis clinic at discharge. For example, a new diagnosis of congestive heart failure or stroke and associated disability might steer the patient toward more preferences related to comfort over life-extending care, whereas receiving a transplant might do the opposite. These issues would be similar across other nondialysis settings, including skilled nursing facilities, transplant centers, and primary and specialty care. Thus, the recommendation to revisit the ESKD Life-Plan when clinically indicated is critical, as is the recognition of these multiple, complex decision points by both patients and providers.^7^ Importantly, events such as hospitalization also present opportunities for providers outside the dialysis care team, such as palliative care providers, to provide information and encourage discussion and documentation of patient preferences.^8^

Finally, in their study, Kazi *et al.*^5^ measured preference-concordant care as a discrete choice between relief of pain and suffering and prolonging life, but this likely creates a false dichotomy. These preferences are not mutually exclusive, and some patients may understandably wish to simultaneously prioritize both improved symptom management and increased life expectancy. Importantly, care preferences and decisions are not limited to the decision to start or withdraw dialysis care—instead, patients have opportunities for decision-making that honor their care preferences during early (*e.g.*, diabetes and hypertension management) and routine (*e.g.*, pursuit of transplant, modality changes, and adherence to medications and fluid intake and diet recommendations) CKD care. Thus, the ESKD Life-Plan should be generalized not only beyond vascular access management but also across the spectrum of CKD and ESKD care (Figure 1).

In summary, the study by Kazi *et al.*^5^ highlights our current challenges in providing preference-concordant care to patients with CKD and patients with ESKD. We propose that a generalized and dynamic version of the ESKD Life-Plan can serve as a blueprint for navigating the multiple, complex decisions CKD and ESKD patients continually face. Therefore, future research that explores the feasibility, utility, and effectiveness of the implementation of such a plan—among specific populations, across the multiple providers that serve this population and at different junctures of patients' CKD trajectories—is warranted. In addition, research to explore the necessary provider support, such as training and education, tools for care coordination, and incentives for the use of a generalized Life-Plan, is needed to improve the concordance of CKD and ESKD care with patient preferences.
